# Supplementary material for: The Memory-CD8+-T-Cell Response to Conserved Influenza Virus Epitopes in Mice Is Not Influenced by Time Since Previous Infection
Source: Vaccines (Basel). 2024 Apr 15;12(4):419. doi: 10.3390/vaccines12040419 (PMC11054904; doi:10.3390/vaccines12040419)
Supplement: Supplementary file 1 [file vaccines-12-00419-s001.zip › vaccines-2939871-supplementary.pdf]

Supplemental Figures

The memory CD8+ T-cell response in mice is not influenced by time since previous infection.

Josien Lanfermeijer[1,2]*, Koen van de Ven[1]*, Marion Hendriks[1], Harry van Dijken[1], Stefanie Lenz[1], Martijn Vos[1], José A.M. Borghans[2], Debbie van Baarle[1,2]*[$], Jorgen de Jonge[1]*[#]
* authors contributed equally
[#] corresponding author

[1] Center for Infectious Disease Control, National Institute for Public Health and the Environment, Bilthoven, the Netherlands
[2] Center for Translational Immunology, University Medical Center Utrecht, the Netherlands
[$] Current Address: Virology & Immunology Research. Dept Medical Microbiology and Infection prevention, University Medical Center Groningen, the Netherlands

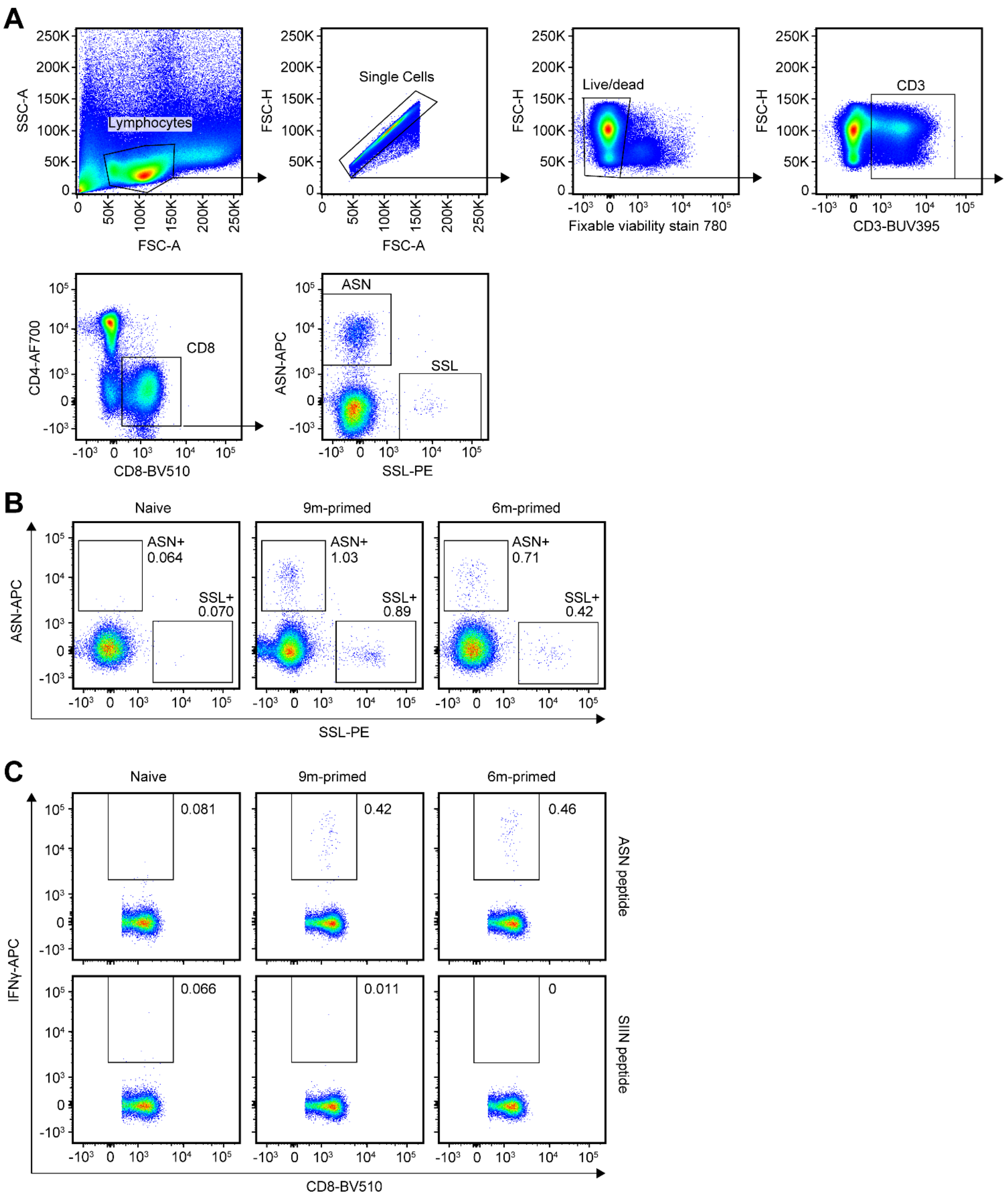

Supplemental Figure S1: Flow cytometry gating strategies. A) Gating strategy of splenocytes for dextramer staining. **B) S**taining of CD8+ T cells with ASN- and SSL peptide loaded dextramers. Mice from day 0 (3 or 6 months after priming) are depicted. **C)** The same gating strategy as depicted in A) was used for IFNγ staining after peptide stimulation, except that in the last panel IFNγ+ cells were gated based on IFNγ-APC vs CD8-BV510. Mice from day 0 (3 or 6 months after priming) are depicted.

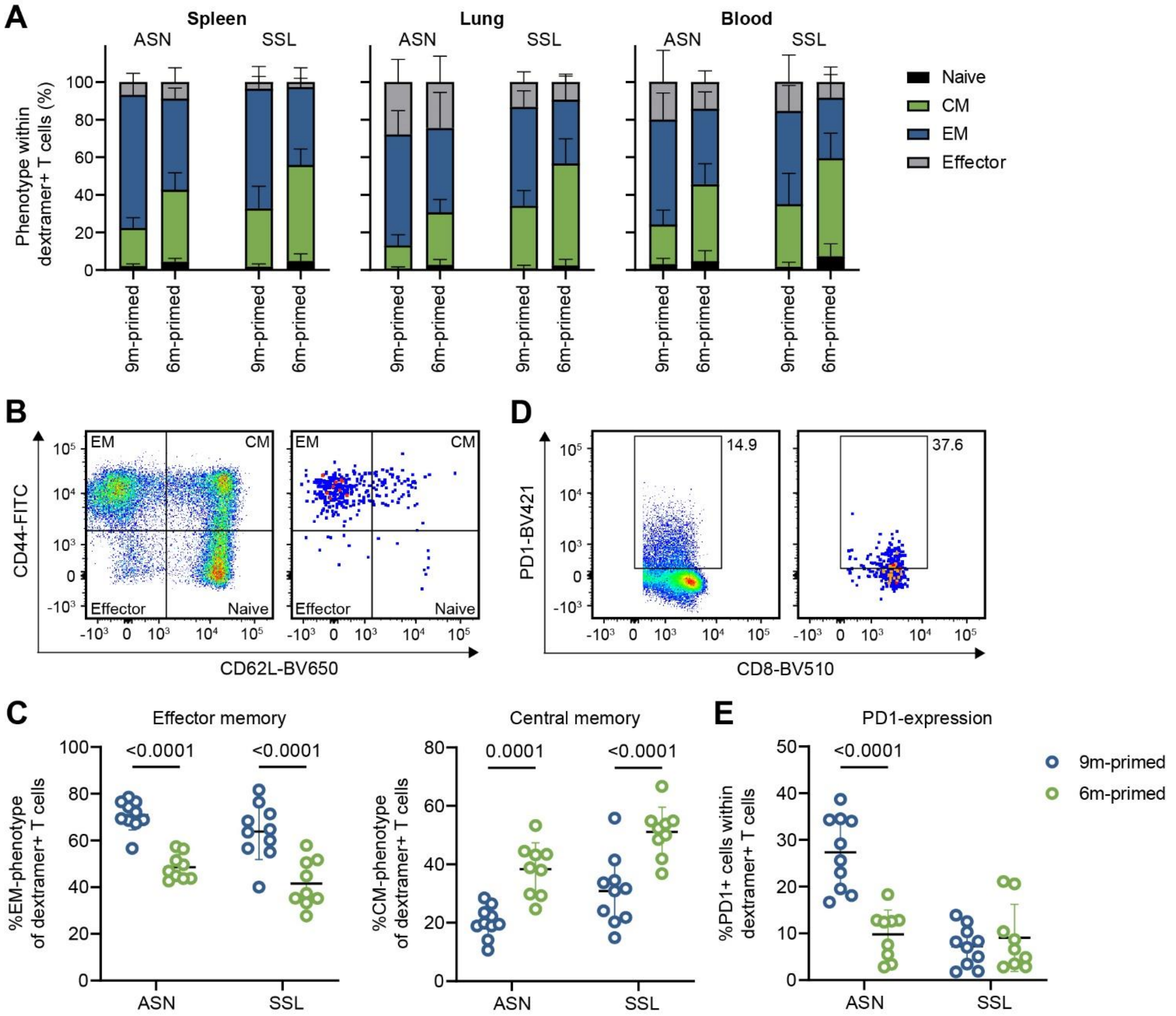

Supplemental Figure S2: Phenotype of the ASN and SSL-specific T cells before booster. A) Fraction of memory subsets based on CD62L and CD44 expression of the ASN-specific and SSL-specific CD8+ T cells in spleen, lung and blood at day 0. Bars depict mean naïve (CD62L+, CD44-), central memory (CM; CD62L+, CD44+), effector memory (EM; CD62L-, CD44+) and effector T cells (CD62L-, CD44-) with standard deviation. **B)** Flow cytometry staining of memory populations. The left panel depicts the whole CD8+ T cell population – on which the gating was based – while the right panel depicts the ASN-dextramer+ CD8+ T-cell population. **C)** Percentage of EM and CM cells within ASN-specific and SSL-specific CD8+ T cells depicted as individual mice with mean and standard deviation. **D)** Flow cytometry staining PD-1 expression. The left panel depicts the whole CD8+ T cell population – on which the gating was based – while the right panel depicts the ASN-dextramer+ CD8+ T-cell population. **E)** Percentage of PD1+ cells within ASN-specific and SSL-specific CD8+ T cells depicted as individual mice with mean and standard deviation. All data shown is from two independent experiments (per timepoint).

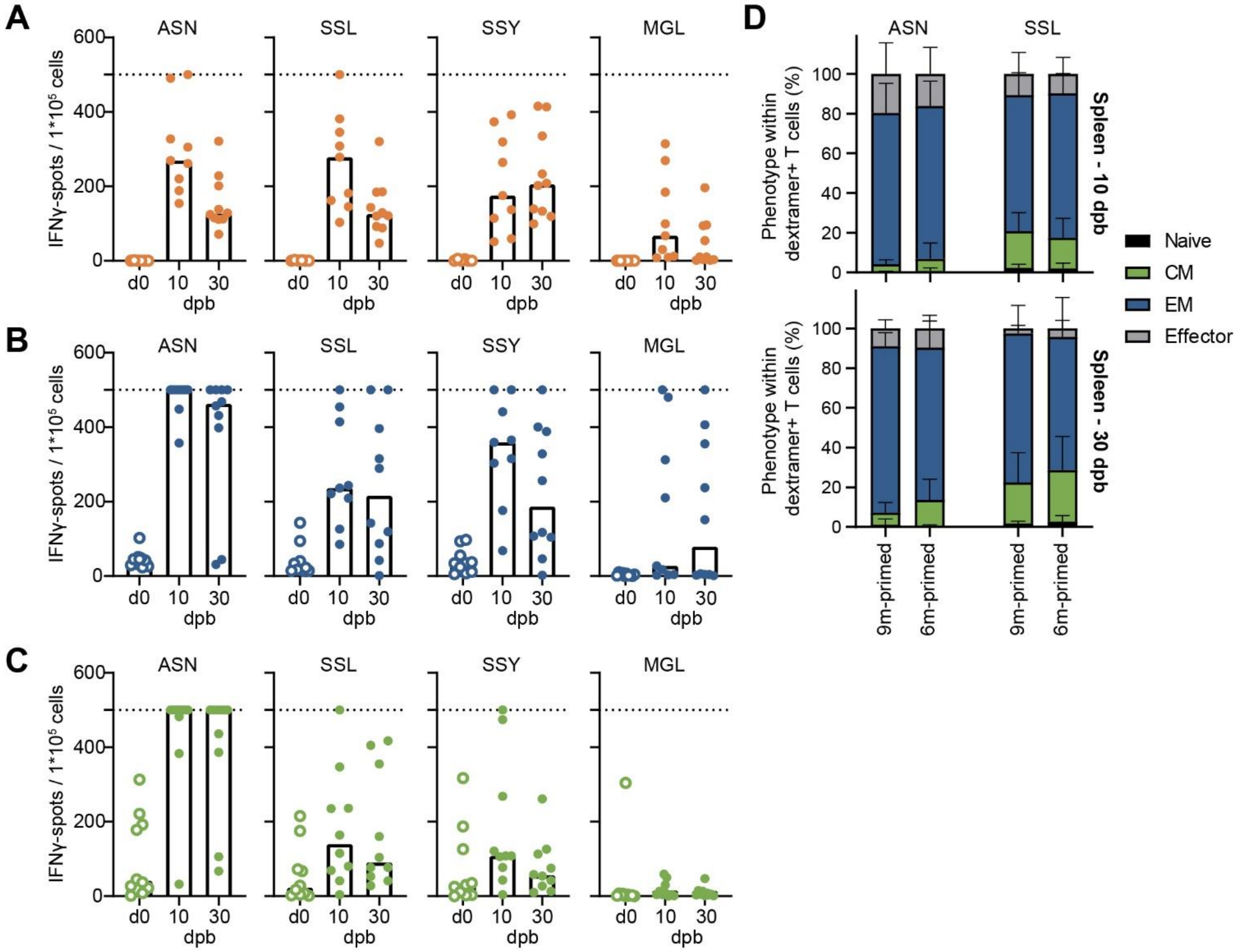

Supplemental Figure S3: Kinetics of T cell response after booster infection. A-C) Cellular responses in IFNγ-ELISpot assays of splenocytes from **A)** previously uninfected mice, **B)** 9m-primed mice and **C)** 6m-primed mice at day 0 (d0) or 10 and 30 days post booster (dpb) after restimulation with IAV specific epitopes. Results are depicted as individual mice (open circles = before booster, closed circles = after booster) with mean (bar). Horizontal dotted lines depict the upper limit of detection of the assay. **D)** Relative size of T-cell subsets based on CD62L and CD44 expression within the ASN-specific and SSL-specific CD8+ T cells in spleen at 10 and 30 dpb. Bars depict mean naïve (CD62L+, CD44-), central memory (CM; CD62L+, CD44+), effector memory (EM; CD62L-, CD44+) and effector T cells (CD62L-, CD44-) with standard deviation. All data shown is from two independent experiments (per timepoint).

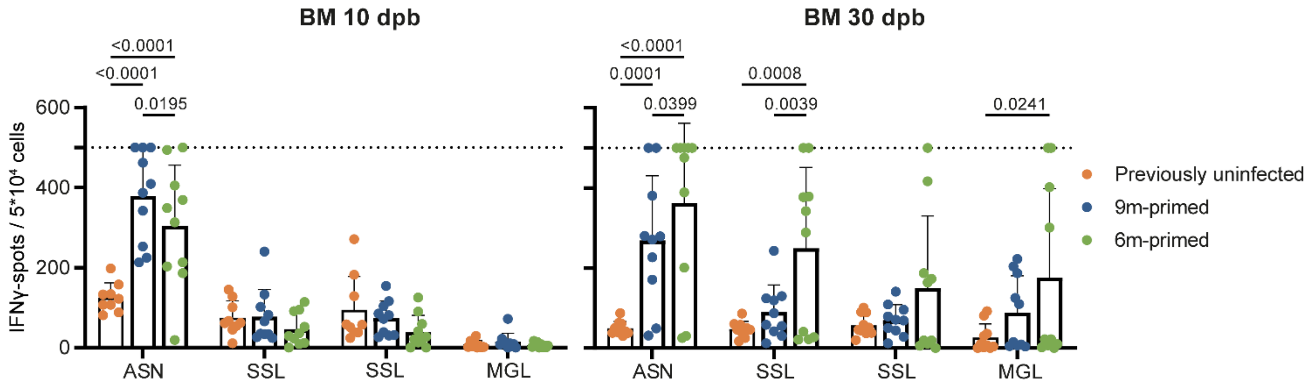

Supplemental Figure S4: IAV-specific T-cell responses at 10 and 30 days post booster (dpb) in the bone marrow. IFNγ-responses measured by IFNγ-ELISpot in bone marrow against the IAV-specific epitopes of previously uninfected mice, and of 9m-primed and 6m-primed mice at 10 and 30 dpb. Results are depicted as individual mice (open circles = before booster, closed circles = after booster) with mean (bar) and standard deviation. All data shown is from two independent experiments (per timepoint).

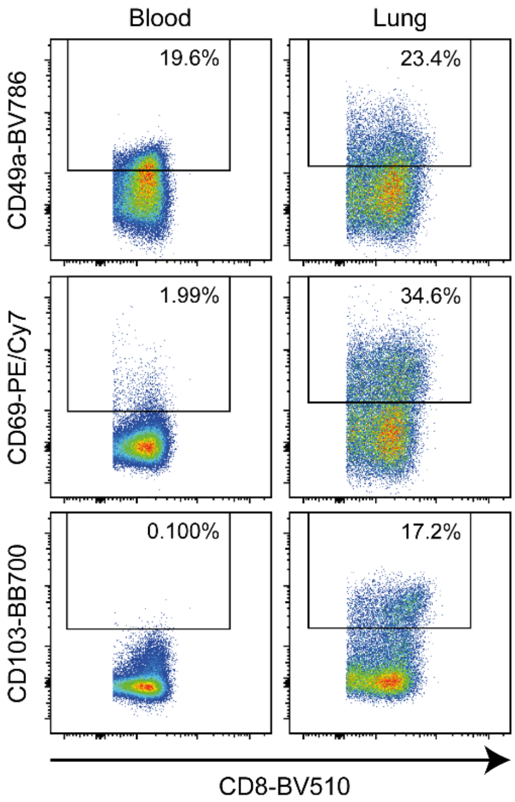

Supplemental Figure S5: Flow cytometry gating of T_{RM} markers. Staining and gating of CD49a, CD69 and CD103 on CD8+ T cells derived from blood and lung of a 6m-primed mouse at 30 days post booster infection.

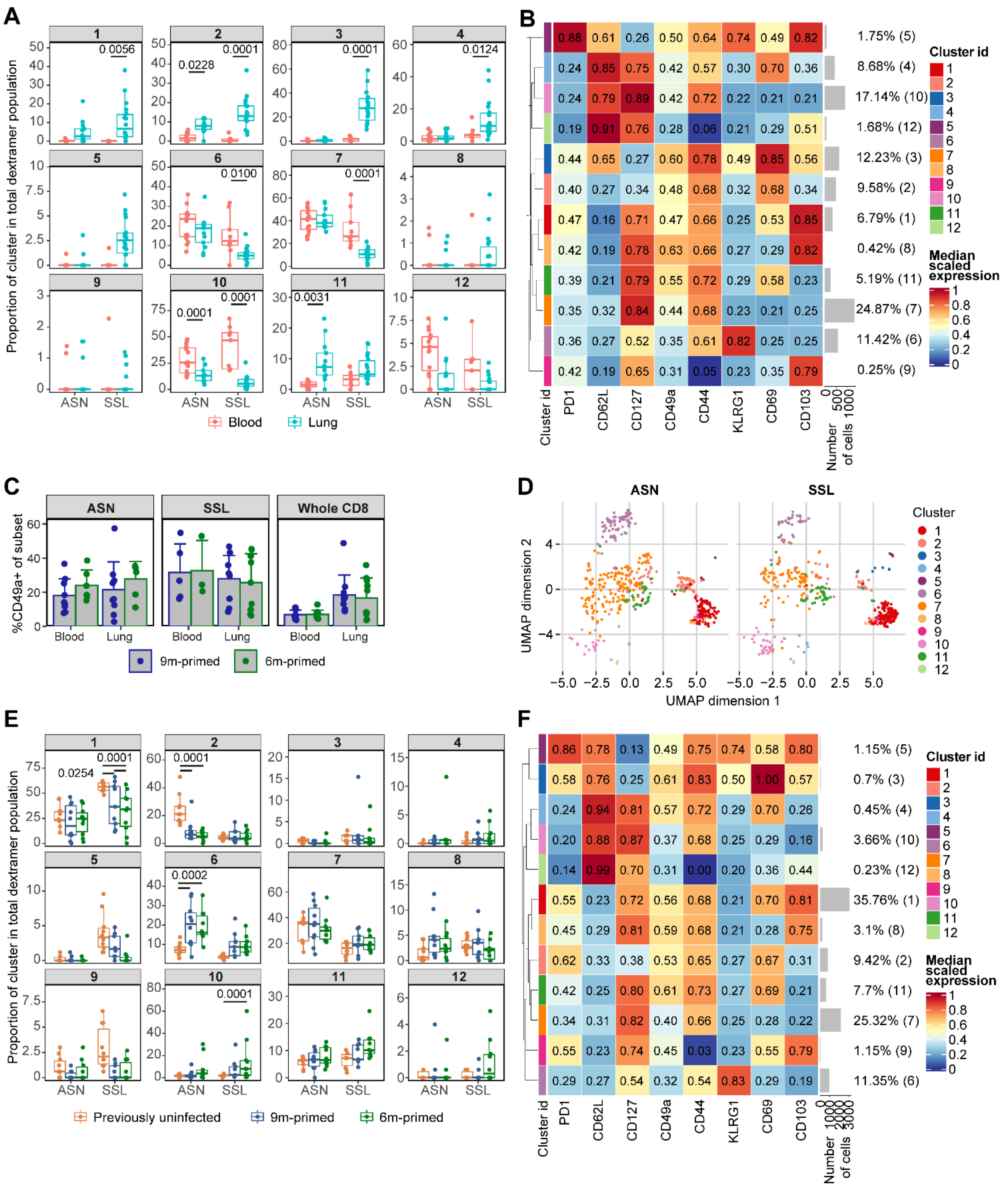

Supplemental Figure S6: uniform manifold approximation and projection (UMAP) analysis of ASN-specific and SSL-specific T cells from blood and lung. **A, B**) UMAP of ASN-specific and SSL-specific CD8+ T cells of blood and lungs from 6m-primed and 9m-primed mice before booster infection (day 0). Data of 6m-primed and 9m-primed mice are combined for this analysis. **A)** Comparison of clusters between blood and lung populations. **B)** Heatmap of 12 clusters generated by UMAP. **C)** Expression of CD49a on ASN-specific, SSL-specific and whole CD8+ T cells from blood and lungs of 6m-primed and 9m-primed mice before booster infection (day 0). **D)** UMAP analysis to compare lung-derived ASN-specific and SSL-specific CD8+ T cells of 6m-primed, 9m-primed and previously uninfected mice 30 dpb. Groups are combined and analyzed separately for ASN-specific and SSL-specific CD8+ T cells. **E, F**) UMAP of ASN-specific and SSL-specific CD8+ T cells from lungs of previously uninfected, 6m-primed and 9m-primed mice after booster infection (day 30). **E)** Comparison of clusters between previously uninfected, 6m-primed and 9m-primed. **F)** Heatmap of 12 clusters generated by UMAP. **A, C, E)** Results are depicted as individual mice (circles) with (A, E) boxplots depicting the median

with the first and third quartiles or (C) bars depicting the mean and standard deviation. All data shown is from two independent experiments (per timepoint).

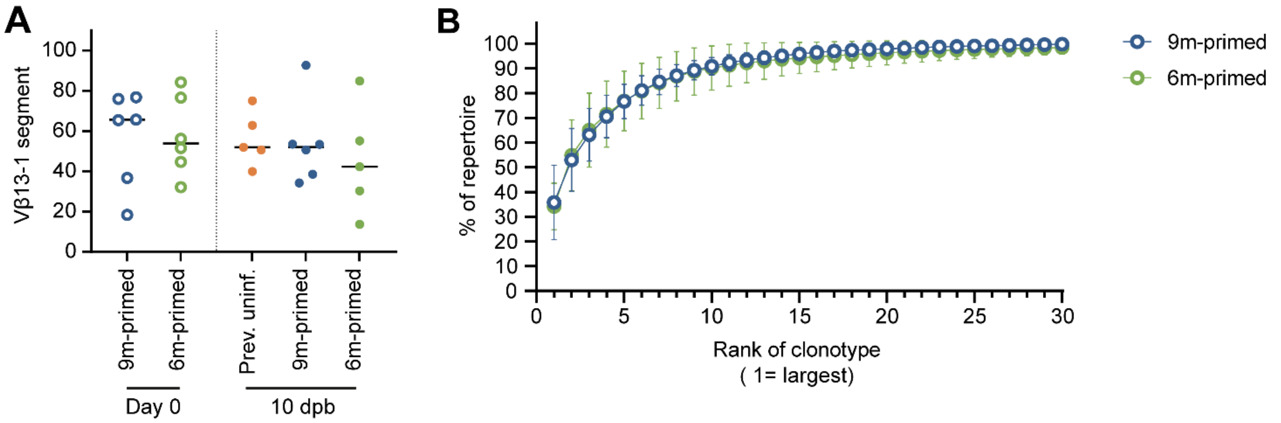

Supplemental Figure S7: Clonal distribution of ASN-specific repertoire before booster. A) Usage of Vβ13-segment within ASN-specific CD8+ T cells isolated from spleen of previously uninfected, 6m-primed and 9m-primed mice at day 0 and 10 days post booster (dpb). Horizontal line depicts the median per group. **B)** Clonal distribution within the TCR repertoire, ranking the top 30 most prevalent clones starting from the largest clone, plotted as the cumulative frequency at day 0. In A, results are depicted as individual mice (open circles = before booster, closed circles = after booster) with mean. In B, results are depicted as average per group (closed circles) with standard deviation. All data shown is from two independent experiments (per timepoint).
